# A Comparative Study of Functional Outcome Following Dynamic Hip Screw and Proximal Femoral Nailing for Intertrochanteric Fractures of the Femur

**DOI:** 10.7759/cureus.23803

**Published:** 2022-04-04

**Authors:** Anil Kumar Prakash, Nagakumar J S, Arun H Shanthappa, Sagar Venkataraman, Amith Kamath

**Affiliations:** 1 Orthopaedics, Sri Devaraj Urs Academy of Higher Education and Research, Kolar, IND

**Keywords:** functional outcome, harris hip score, proximal femoral nailing, dynamic hip screw, intertrochanteric fractures

## Abstract

Introduction

Intertrochanteric fractures are common in the old age group. The goal/aim of the treatment for intertrochanteric fractures will be to nearly restore pre-injury condition as early as it is possible. Dynamic hip screw (DHS) and proximal femoral nailing (PFN) have been the two standard treatment methods used for treating these kinds of fractures. The main goal of this proposed study was to compare functional outcomes of two available fixation devices for inter-trochanteric fracture using Harris hip scoring. The aim of this study is to compare the functional outcome of the DHS and PFN for the treatment of Intertrochanteric hip fractures achieved by the patient based on Harris hip score.

Methods and materials

The clinical methodology for the study consists of 46 cases of Inter-trochanteric fractures of femur that meet the inclusion criteria of patients aged above 45 years diagnosed with closed intertrochanteric fractures that are less than three weeks duration who were able to walk prior to fracture and exclusion criteria, admitted to R L Jalappa Hospital, Tamaka, Kolar between November 2019 and November 2021. The patients were divided into two groups, group A treated with DHS and group B treated with PFN and followed up at six weeks, 12 weeks, and 24 weeks based on the functional outcome on the 24^th^ week using Harris hip score.

Results

A total of 46 patients were included in the study. The mean age in Group DHS was 61.09 ± 11.69 and in Group PFN was 65 ± 14.98. In the group of DHS, nine out of 23 patients were male and 14 out of 23 patients were female patients. In a group of PFN, 12 out of 23 patients were male and 11 out of 23 patients were female. The mean six weeks score in Group DHS was 34.43 ± 3.23 out of 100 and in Group PFN was 34.35 ± 2.5 out of 100. The mean Harris hip score in Group DHS was 84.3 ± 7.68 out of 100. The mean Harris hip score in Group PFN was 89.26 ± 6.53 out of 100. In Group DHS, 52.17% had injuries on the left side and 47.83% had on the right side. In Group PFN, 39.13% had Injury on the left side and 60.87% had on the right side. In Group DHS, results were excellent in 34.78% (eight patients), good in 43.48% (10 patients), fair in 17.39% (four patients out of 23 patients), and poor in 4.35% (one patient). In Group PFN, results were excellent in 56.52% (13 patients), good in 34.78% (eight patients), and fair in 8.70% (two patients).

Conclusion

From the study, it can be concluded that PFN had a better outcome in intertrochanteric fractures compared to DHS. The highest percentage of subjects in the PFN group had excellent to a good outcome and none of them had poor outcomes when compared to the DHS group. PFN group had higher scores of Harris hip score at 12 weeks, 24 weeks, and at the end of follow-up.

## Introduction

Intertrochanteric fractures are very common in the old age group, but infrequent in the younger age group. In intertrochanteric fractures treated conservatively which healed with vicious callus, coxa-vara deformity is frequently observed, resulting in lower limb shortening and limb flaccidity [1]. Multiple surgical procedures with multiple different implants have been described in the literature and used for the treatment of intertrochanteric fractures. Little possible attention has been paid to these kinds of fractures in the past because they arise from porous bone with an excellent and rich blood-supply and can heal without active intervention. Conservative treatment, however, resulted in a vicious callus with varus, external rotation with shortening resulting in the short limp gait of walking and a high mortality rate due to the complications when lying down and prolonged immobilization. The goal/aim for the treatment for intertrochanteric fractures will be to nearly restore pre-injury condition as early as possible. This has led to internal fixation to increase the patient comfort by facilitating nursing care, reducing hospitalization, early mobilization, and reducing complications. Problems in treating this fracture are the instability and fixation complications that will result from the treatment of the intertrochanteric fractures. Stability is the ability of an internally attached fracture to withstand gravity and muscle forces acting around it and cause the fracture to undergo varus displacement. Other contributing factors that might contribute mostly to fixation failure are some intrinsic factors such as the fracture reduction of the fractures and osteoporosis and some extrinsic contributing factors such as implant of choice and insertion technique [2].

The implant type used will affect the final outcome and the complication of that fixation that might accompany the fracture and its fixation. Dynamic hip screw (DHS), and sliding plate device, is already widely used for fixation. However, if weight bearing is started early, especially in the compound and comminuted fractures, the device may have a tendency to penetrate or retract through the head. The proximal femoral nailing (PFN) is the intramedullary device that has commonly been reported to have benefited in such fractures because its placement is close to its mechanical-axis of the body and thus it reduces the lever arm aspect on the implant. In addition, they also take very little time to insert with little blood loss, allow early weight-bearing movement post-surgery, and result in less short long-term follow-up. The main aim/goal of this proposed study was to compare functional outcomes of two available fixation devices for intertrochanteric fracture and if anyone device can have an advantage over the other in terms of the patient's ultimate functional outcome using Harris hip scoring.

## Materials and methods

The clinical methodology for the study consists of 46 cases of intertrochanteric fractures of femur that meet the inclusion criteria and exclusion criteria, admitted to R L Jalappa Hospital, Tamaka, Kolar between November 2019 and July 2021. Prior to the start of the study, Sri Devaraj Urs Medical College Institutional ethics committee clearance was obtained with approval number SDUMC/KLR/IEC/156/2019-2020. The inclusion criteria consist of patients aged above years diagnosed with closed intertrochanteric fractures that are less than three weeks duration who were able to walk prior to fracture. Excluding patients with malignancy, neurological, psychiatric illness, and patients associated with co-morbid conditions like uncontrolled diabetes mellitus, uncontrolled hypertension, hyperthyroidism, patients with active infections of hip joints from the study.

Once the patient was admitted, a detailed history was elicited and a head-to-toe patient examination was done. The patient’s radiographs of the pelvis with bilateral hip joints in AP view were taken. The confirmed diagnosis of the patient was made by clinical and radiological examination. Static traction was then applied in the form of skin traction or skeletal traction. The required information given by the patient was recorded as per the proforma. Patients were taken for surgery after obtaining written informed consent about the nature and complications of the surgery. The patients were grouped into DHS and PFN groups based on simple randomization. The selected patients were taken up once clearance for surgery was taken from the anesthetist and physician/cardiologist if required.

All patients were prophylactically started on third-generation cephalosporins (inj ceftriaxone 1 g IV, half to one hour prior to the start of surgery. All patients received postoperative injectable antibiotics, intravenous cephalosporins for five days, followed by oral antibiotics until the sutures were removed. Static quadriceps strengthening exercises were started on the second or third postoperative day. The drain if placed was later removed after the third postoperative day. The sutures were removed after 10 to 14 days. The patients were mobilized without support as soon as localized pain or general patient condition permitted. Partial support was started six weeks after clinical and radiological assessment and full support was performed 12 weeks after the assessment. And recalled after six months for the final follow-up and assessment using Harris hip score (HHS).

The final result is based on the HHS, which includes areas like pain, a function of the joint, absence/presence of deformity, and range of movements. The pain domain measures pain severity and its effect on activities and needs for pain medication. The function part of the domain consists of daily activities like (staircase use, using public transportation, sitting, tying/managing shoes and socks) and gait (limp, support needed, and walking distance). Deformity takes these factors into accounts such as hip flexion, adduction, internal rotation, and extremity length discrepancy. Range of motion measures hip flexion, abduction movement, adduction, external and internal rotation. The HHS score gives a maximum of 100 points. Pain receives 44 points, function 47 points, range of motion 5 points, and deformity 4 points. A function is subdivided into activities of daily living (14 points) and gait (33 points). The higher the HHS, the less the dysfunction. A total score of <70 scores are considered a poor result; 70-80 is considered fair, 80-90 is good, and 90-100 is excellent.

Data were entered into a Microsoft Excel datasheet and was analyzed using SPSS 22 version software (IBM SPSS Statistics, Somers NY, USA). Categorical data was represented in the form of frequencies and proportions. The Chi-square test was used as a test of significance for qualitative data. Continuous data were represented as mean and standard deviation. Independent t-test was used as a test of significance to identify the mean difference between two quantitative variables and qualitative variables, respectively [3-5]. Graphical representation of data: MS Excel and MS word was used to obtain various types of graphs such as bar diagram and line diagram. P-value (probability that the result is true) of <0.05 was considered as statistically significant after assuming all the rules of statistical tests. Statistical software, MS Excel, SPSS version 22, was used to analyze data.

## Results

There was no significant difference in gender distribution between the two groups (Table 1). There was a significant difference in the mode of injury distribution between the two groups (Table 1). There was not much significant difference in Boyd and Griffin classification distribution between the two groups (Table 1).

**Table 1:** Sociodemographic data DHS = dynamic hip screw; PFN = proximal femoral nail

Characteristics	DHS group	PFN group
Mean (SD) age	61.09	65
Gender		
Male	9(39.13%)	12(47.83%)
Female	14(60.87%)	11(52.17%)
Side		
Left	12(52.17%)	9(39.13%)
Right	11(47.83%)	14(60.87%)
Mode of injury		
RTA	1(4.35%)	6(26.09%)
Self-fall	5(21.74%)	17(73.91%)
others	17(73.91%)	0(0.00%)
Boyd and Griffin classification		
Type-1	6(26.09%)	9(39.13%)
Type-2	13(56.52%)	8(34.78%)
Type-3	2(8.70%)	5(21.74%)
Type-4	2(8.70%)	1(4.35%)

The mean six weeks score in Group DHS was 34.43 ± 3.23 out of 100 and in Group PFN was 34.35 ± 2.5 out of 100. There was not much significant difference in the mean six weeks comparison between the two groups. The mean 12 weeks score in Group DHS was 54.65 ± 2.69 out of 100 and in Group PFN was 62.17 ± 5.99 out of 100. There was a significant difference in the mean 12 weeks comparison between the two groups. The mean 24 weeks score in Group DHS was 84.3 ± 7.68 out of 100 and in Group PFN was 89.26 ± 6.53 out of 100. There was a significant difference in the mean 24 weeks comparison between the two groups (Table 2). Mean HHS in Group DHS was 84.3 ± 7.68 out of 100. Mean HHS in Group PFN was 89.26 ± 6.53 out of 100. There was a significant difference in the mean HHS ( /100) comparison between the two groups (Table 2).

**Table 2:** Mean score comparison between the DHS and PFN group at the six, 12 and 24-week follow up DHS = dynamic hip screw; PFN = proximal femoral nail; SD = standard deviation

	Group				P-value
	DHS		PFN		
	Mean	SD	Mean	SD	
6 weeks	34.43	3.23	34.35	2.5	0.919
12 weeks	54.65	2.69	62.17	5.99	< 0.001*
24 weeks	84.3	7.68	89.26	6.53	0.023*

In Group DHS, results were excellent in 34.78% (eight patients out of 23 patients), good in 43.48% (10 patients out of 23 patients), fair in 17.39% (four patients out of 23 patients), and poor in 4.35% (one patient out of 23 patients). In Group PFN, results were excellent in 56.52% (13 patients out of 23 patients), good in 34.78% (eight patients out of 23 patients), and fair in 8.70% (two patients out of 23 patients). There was not much significant difference in results distribution between the two groups (Table 3). Functional outcome is interpreted based on HHS at the end of 24 weeks, <70 = poor result; 70-80 = fair, 80-90 = good, and 90-100 = excellent.

**Table 3:** Functional outcome distribution between DHS and PFN groups DHS = dynamic hip screw; PFN = proximal femoral nail

		Group			
		DHS		PFN	
		Count	Column N %	Count	Column N %
Result	Excellent	8	34.78%	13	56.52%
	Good	10	43.48%	8	34.78%
	Fair	4	17.39%	2	8.70%
	Poor	1	4.35%	0	0.00%

Functional outcome

**Figure 1:**
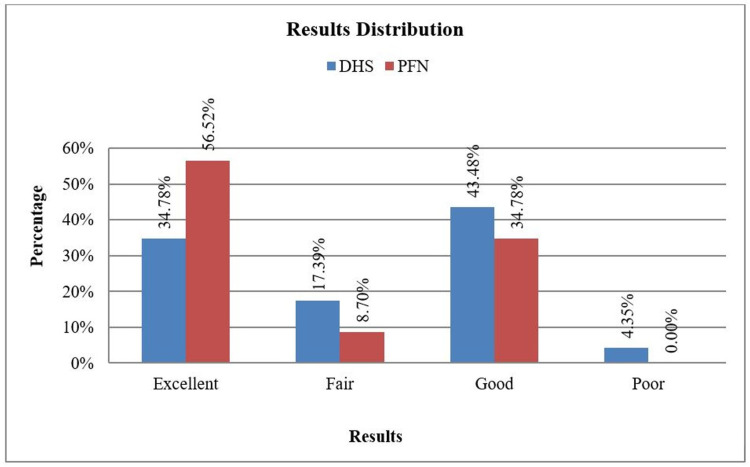
Bar diagram showing the functional outcome of the DHS and the PFN group based on Harris hip score DHS = dynamic hip screw; PFN = proximal femoral nail

Out of the 46 cases in the study, out of the 46 cases which had an excellent outcome 23 were treated with PFN, and 23 were treated by DHS. Thirteen cases treated by PFN and eight cases treated by DHS had an excellent result. Eight cases treated by PFN and 10 cases treated by DHS had a good result. A fair result was recorded in two cases treated by PFN and four cases treated by DHS. One patient who was treated by DHS had a poor functional outcome (Figure 1).

**Figure 2:**
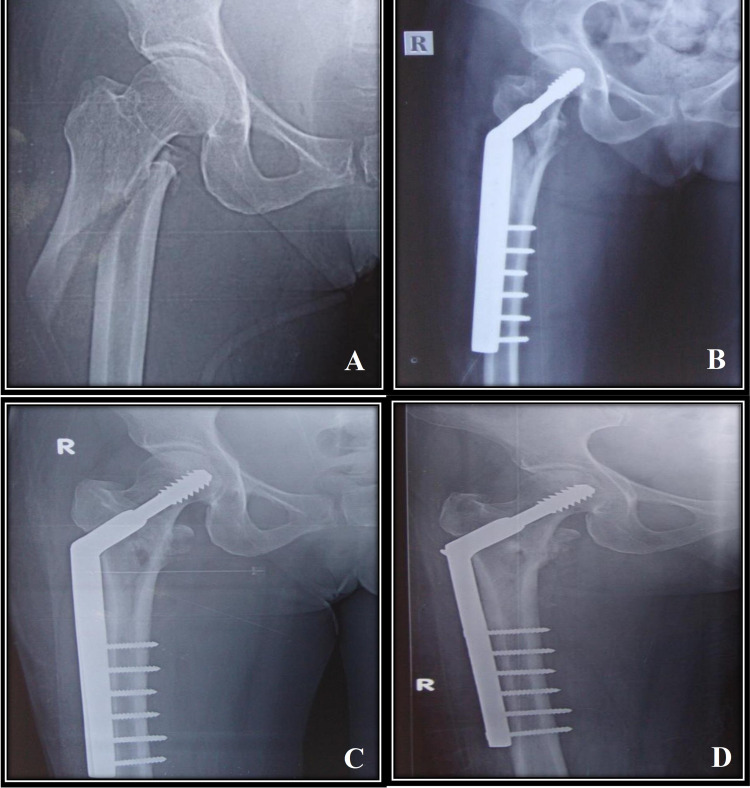
Pre-operative and post-operative follow-up radiographs of an intertrochanteric fracture treated with DHS fixation (A) Pre-operative radiograph. (B) Immediate post-operative radiograph. (C) 12-week follow-up radiograph. (D) 24-week follow-up radiograph. DHS = Dynamic hip screw

**Figure 3:**
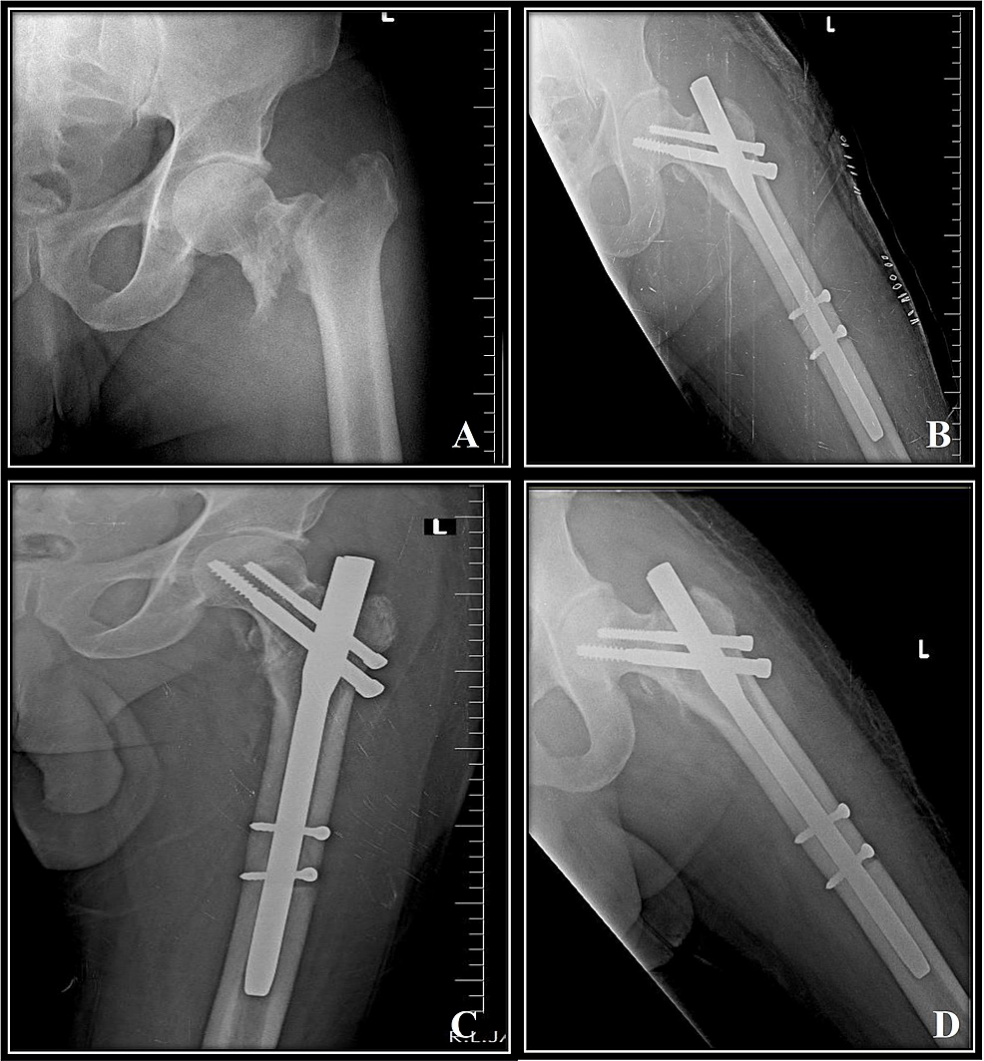
Pre-operative and post-operative follow-up radiographs of an intertrochanteric fracture treated with PFN fixation (A) Pre-operative radiograph. (B) Immediate post-operative radiograph. (C) 12-week follow-up radiograph. (D) 24-week follow-up radiograph. PFN = Proximal femoral nail

Pre-operative, immediate post-operative, 12 weeks and 24 weeks follow up radiographs of a case treated with DHS fixation (Figure 2) and PFN fixation (Figure 3). There were two cases with superficial surgical site infection in the DHS group which were treated with culture sensitivity-based antibiotic usage and infection subsided. Limb length discrepancy of 1-2 cm was noted in three patients (two patients in the DHS group and one patient in the PFN group) which were treated with shoe raise and gait training. No deformities were noted in the study. No periprosthetic fractures were noted in the study.

 

## Discussion

Fractures of the intertrochanteric region of the femur have been recognized as a major challenge by the Orthopedic community, not just only for achieving fractures union, but for also restoration of optimal function in the least short possible time with very minimal complications. The aim of fracture management accordingly has drifted to achieving very early mobilization, rapid rehabilitation, and quick return of the individuals to pre-morbid home and work-like environment as a functionally and psychologically independent unit.

Operative/surgical treatment in the form of internal fixation permits very early rehabilitation and offers the best chances of functional recovery, and hence has become the gold standard treatment of choice for virtually all fractures in the intertrochanteric region. Among the various types of implants available, i.e., fixed nail plate devices, sliding nails or the screw plates, and intramedullary devices, the compression hip-screw is most commonly used (still remains the gold standard) but recently surgical techniques of closed intramedullary nailing have gained very high popularity.

In this study, an attempt was made to survey, evaluate, document, and quantify our success in the management of such individuals by using the PFN and the DHS implants and then comparing the results in these two groups. The study was conducted on 46 patients (23 cases by PFN and 23 cases by DHS) of proximal femoral fractures attending outpatient/causality department of Orthopedics, R L Jalappa Hospital, constituent hospital of SDUMC, SDUAHER, deemed to be a university for a period of two years.

Profile of subjects in the study

In the study the factors such as age, gender, side of injury, mode of injury, and type of fracture were matched to eliminate selection bias.

Age distribution

In the present study, the mean age in Group DHS was 61.09 ± 11.69 and in Group PFN was 65 ± 14.98. This signifies that patients from the age group are involved in low-energy trauma like falls (fall at home). The reason why the trochanteric region is the most common site of senile osteoporosis as age advances. The hip joint being a major joint n the mechanism of weight-bearing, this already weakened part cannot withstand any sudden abnormal stress. The space between bony trabeculae is enlarged and loaded with fat, whilst unsheathing compact tissue is thinned out and calcar is atrophied. Due to the early fixation of such intertrochanteric fractures and early mobilization, these patients could gain a full range of movements at an early date with very minimal loss of productivity. In a study of 40 patients conducted by Amandeep et al. [6], the mean age in the DHS group was 60.3, and that in the PFN group was 56.85. In another study of 52 patients conducted by Kushal et al. [7], the mean age in the DHS group was 65, and that in the PFN group was 70.2. Our study has statistics similar to that of Amandeep et al. [6].

Gender distribution

In Group DHS, 39.13% were male and 60.87% were female. In Group PFN, 52.17% were male and 47.83% were female. Hence a female predominance was seen for intertrochanteric fractures. The following reasons were given by Cleveland et al. [8] for female preponderance. Females have a slightly wider pelvis with a tendency to have coxa vara. They are usually less active and are more prone to senile osteoporosis. In the comparative study by Pan et al. [9], the males comprised 75% of the study group. In his study of 80 cases, Shakeel et al. [10] found that 66% of the study group was males. Zhao et al. [11] describe the male incidence at 40%. In his study of 80 cases, Gill et al. [12] found that males comprised only 32% of the study group. Our study has findings similar to that of Gill et al. and Zhao et al. with female preponderance [11].

In Group DHS, 52.17% had an injury on the left side and 47.83% had on the right side. In Group PFN, 39.13% had Injury on the left side and 60.87% had on the right side. 

In Group DHS, the mode of Injury was a road traffic accident (RTA) in 4.35%, self-fall in 21.74%, and trivial fall in 73.91%. In Group PFN, the mode of injury was RTA in 26.09%, and self-fall in 73.91%. This can be attributed to the following factors, listed by Cummings and Nevitt in 1994 [13]. Insufficient shielding reflexes to reduce fall energy below a certain critical threshold. Insufficient local shock absorbers, for example. muscle and fat around the hip. Insufficient bone strength in the hip due to osteoporosis or osteomalacia. Young patients with intertrochanteric or sub-trochanteric fractures have suffered trauma as a result of a traffic accident or a fall from a height, reflecting the need for high-velocity trauma to cause fractures in young people. Keneth J. Koval and Joseph D. Zuckerman noted that 90% of hip fractures in older people are the result of a simple fall. Hip fractures in young adults are often the result of high-energy trauma such as motor vehicle collisions or falls from a height. In his study of 30 cases, Mundla et al. [14] described 70% of cases as a result of trivial falls while 23% was due to RTA. Jonnes et al. [15] conducted a study on 30 cases where they described 77% of cases as a result of trivial falls while the remaining 23% was due to RTA. In his study on 80 patients, Gill et al. [12] concluded that 66% of cases were a result of trivial falls while the remaining were due to RTA. Our study also highlights that trivial fall is perhaps the important contributing cause of IT fractures (Table 4).

**Table 4:** Mechanism of injury comparison between other studies RTA = road traffic accident

Study	Trivial fall	RTA
Mundla et al. [14]	70%	23.3%
Jonnes et al. [15]	77%	23%
Gill et al. [12]	66%	34%

Type of fracture

We have classified Intertrochanteric fracture based on Boyd and Griffin classification. In Group DHS, 26.09% had Type -1, 56.52% had Type - 2, 8.70% had Type - 3 and 8.70% had Type - 4. In Group PFN, 39.13% had Type -1, 34.78% had Type - 2, 21.74% had Type - 3 and 4.35% had Type - 4. According to Windolf et al. [16], intertrochanteric fractures are considered stable or unstable depending on the integrity of the posteromedial cortex. Fractures with intact posteromedial cortex are considered stable fractures, while fractures with loss of the posteromedial cortex are considered unstable fractures. The posteromedial cortex is primarily the lesser trochanter.

In the present study at six weeks score in Group DHS was 34.43 ± 3.23 and in Group PFN was 34.35 ± 2.5. There was no significant difference in the mean six weeks comparison between the two groups. The mean 12 weeks score in Group DHS was 54.65 ± 2.69 and in Group PFN was 62.17 ± 5.99. There was a significant difference in the mean 12 weeks comparison between the two groups. The mean 24 weeks score in Group DHS was 84.3 ± 7.68 and in Group PFN was 89.26 ± 6.53. There was a significant difference in the mean 24 weeks comparison between the two groups. In a study of 40 patients conducted by Amandeep et al. [16], the mean HHS in the DHS group was 83.75, and that in the PFN group was 84.4. In his study of 80 cases, Shakeel et al. [11] found that the mean HHS in the DHS group was 73.73 while in the PFN group, it was 83.5. In a study of 60 patients conducted by Sharma et al. [17], the mean HHS in the DHS group was 88.7, and that in the PFN group was 82.2 (Table 5).

**Table 5:** Mean Harris hip score comparison between other studies DHS = dynamic hip screw; PFN = proximal femoral nail

	Mean Harris hip score	
	DHS	PFN
Amandeep et al. [6]	83.75	84.4
Shakeel et al. [10]	73.73	83.5
Anmol Sharma et al. [17]	88.7	82.2
Present study	84.3	89.26

Functional outcome

In Group DHS, results were excellent in 34.78%, fair in 17.39%, good in 43.48%, and poor in 4.35%. In Group PFN, results were excellent in 56.52%, fair in 8.70%, and good in 34.78%. The range of movement calculated by the HHS system treated by both the implants, i.e., PFN and DHS was good and was almost the same. The range of movements namely flexion, extension, external and internal rotation was good in most cases, excellent in a few. Very few there were fair results. The fair result was attributed to other associated factors namely a long interval between trauma and surgery and the development of postoperative infection. Kushal et al. [7] in the study of 52 patients noted that in the DHS group, excellent results were seen in six (23%), good results seen in five (19%), fair results seen in 13 (50%), and poor results seen in two (8%). In the PFN group, excellent results were seen in four (15%), good results seen in 14 (54%), fair results seen in seven (27%), and poor results seen in one (4%).

Harish et al. [18] in the study of 30 patients noted that in the DHS group, excellent results were seen in six (50%), good results seen in two (13.33%), fair results seen in two (13.33%), and no poor results were seen. In the PFN group, excellent results were seen in eight (72.73%), good results seen in one (9.1%), fair results seen in one (9.1%), and no poor results were seen (Table 6).

**Table 6:** Functional outcome in our studies DHS = dynamic hip screw; PFN = proximal femoral nail

Functional outcome	DHS	PFN
Excellent	6(50%)	8(72.73%)
Good	2(13.33%)	1(9.1%)
Fair	2(13.33%)	1(9.1%)
Poor	2(13.33%)	1(9.1%)

Gill et al. [12], in his comparative study of 80 patients using the Locking DHS and PFN, noted that in the DHS group, excellent results were seen in six (15%), good results seen in 14 (35.0%), fair results seen in 12 (30.0%), and poor results seen in eight (20.0%). In the PFN group, excellent results were seen in eight (20.0%), good results seen in 130 (75.0%), fair results seen in two (5.0%), and no poor results were seen (Table 7).

**Table 7:** Functional outcome in other studies DHS = dynamic hip screw; PFN = proximal femoral nail

	Functional outcome		Total
	DHS	PFN	
Excellent	6(15.0%)	8(20.0%)	12(27.272%)
Good	14(35.0%)	30(75.0%)	30(68.181%)
Fair	12(30.0%)	2(5.0%)	0(0.0%)
Poor	8(20.0%)	0(0.0%)	2(4.545%)
Total	20(100.0%)	24(100.0%)	44(100.0%)

In the present study in both groups, two cases with superficial surgical site infection in the DHS group might have been because of the longer incision exposure to open pathogens during surgery. Shakeel et al. [11] and Gill et al. [12] noted a high incidence of superficial infection in the DHS group which they attributed to the lengthier incision associated with DHS. This is similar to the findings of our study. Limb length discrepancy of 1-2 cm was noted in three patients (two patients in the DHS group and one patient in the PFN group), which is also similar to the study conducted by Amandeep et al. [6].

Limitations of the study were that long-term complications were not studied, a smaller sample size due to the ongoing COVID-19 pandemic, factors affecting the outcome were not studied in both groups, e.g., the influence of the surgeon’s expertise and the cost of both the procedures were not compared.

## Conclusions

From the study based on the functional outcome derived from Harris Hip Score, it can be concluded that PFN had a better outcome in intertrochanteric fractures compared to DHS fixation. This was concluded based on the final outcome, range of movements, and HHS. The highest percentage of subjects in the PFN group had excellent to a good outcome and none of them had poor outcomes when compared to the DHS group. PFN group had higher scores of HHS at 12 weeks, 24 weeks and at the end of follow-up. PFN has a faster recovery and better functional outcome in all types of intertrochanteric fracture with fewer complications.

## References

[1] Pajarinen J, Lindahl J, Michelsson O, Savolainen V, Hirvensalo E (2005). Pertrochanteric femoral fractures treated with a dynamic hip screw or a proximal femoral nail. A randomised study comparing post-operative rehabilitation. J Bone Joint Surg Br.

[2] Kaufer H, Matthews LS, Sonstegard D (1974). Stable fixation of intertrochanteric fractures. J Bone Joint Surg Am.

[3] Gao H, Bai X, Chen W (2020). Clinical and functional comparison of dynamic hip screws and intramedullary nails for treating proximal femur metastases in older individuals. Chin J Cancer Res.

[4] Dakhale GN, Hiware SK, Shinde AT, Mahatme MS (2012). Basic biostatistics for post-graduate students. Indian J Pharmacol.

[5] Sunder Rao PSS, Richard J (2006). An Introduction to biostatistics. A Manual for Students in Health Sciences.

[6] Bakshi AS, Kumar P, Brar BS (2018). Comparative study between DHS and PFN in intertrochanteric fractures of femur. Int J Orthop Sci.

[7] Parikh KN, Parmar C, Patel M (2018). Functional and radiological outcome of proximal femoral nailing versus dynamic hip screw in unstable intertrochanteric femur fractures. Int J Res Orthop.

[8] Cleveland M, Bosworth D, Thompson F (1959). A ten-year analysis of intertrochanteric fractures of the femur. J Bone Joint Surg Am.

[9] Huang X, Leung F, Xiang Z, Tan PY, Yang J, Wei DQ, Yu X (2013). Proximal femoral nail versus dynamic hip screw fixation for trochanteric fractures: a meta-analysis of randomized controlled trials. Sci World J.

[10] Zhao C, Liu DY, Guo JJ, Li LP, Zheng YF, Yang HB, Sun JH (2009). Comparison of proximal femoral nail and dynamic hip screw for treating intertrochanteric fractures (Article in Chinese). Zhongguo Gu Shang.

[11] Qidwai SA, Singh R, Mishra AN (2019). Comparative study of functional outcome of the intertrochanteric fracture of femur managed by dynamic hip screw and proximal femoral nail. Nat J Clin Orthop.

[12] Gill SPS, Mittal A, Raj M, Singh P, Kumar S, Kumar D (2017). Dynamic hip screw with locked plate VRS proximal femoral nail for the management of intertrochanteric fracture: a comparative study. Int J Orthop Sci.

[13] Cummings SR, Nevitt MC (1994). Non-skeletal determinants of fractures: the potential importance of the mechanics of falls. Study of Osteoporotic Fractures Research Group. Osteoporos Int.

[14] Mundla MKR, Shaik MR, Buchupalli SR (2018). A prospective comparative study between proximal femoral nail and dynamic hip screw treatment in trochanteric fractures of femur. Int J Res Orthop.

[15] Jonnes C, Sm S, Najimudeen S (2016). Type II intertrochanteric fractures: proximal femoral nailing (PFN) versus dynamic hip screw (DHS). Arch Bone Jt Surg.

[16] Windolf J, Hollander DA, Hakimi M (2005). Pitfalls and complications in the use of the proximal femoral nail. Langenbecks Arch Surg.

[17] Sharma A, Sethi A, Sharma S (2018). Treatment of stable intertrochanteric fractures of the femur with proximal femoral nail versus dynamic hip screw: a comparative study. Rev Bras Ortop.

[18] Harish K, Paleti ST, Kumar RN (2019). A comparative study between DHS and PFN for the treatment of IT fractures. Nat J Clin Orthop.

